# Early Detection of Pandemic (H1N1) 2009, Bangladesh

**DOI:** 10.3201/eid1801.101996

**Published:** 2012-01

**Authors:** Eduardo Azziz-Baumgartner, Mustafizur Rahman, Abdullah Al Mamun, Mohammad Sabbir Haider, Rashid Uz Zaman, Polash Chandra Karmakar, Sharifa Nasreen, Syeda Mah-E Muneer, Nusrat Homaira, Doli Rani Goswami, Be-Nazir Ahmed, Mohammad Mushtuq Husain, Khondokar Mahbuba Jamil, Selina Khatun, Mujaddeed Ahmed, Apurba Chakraborty, Alicia Fry, Marc-Alain Widdowson, Joseph Bresee, Tasnim Azim, A.S.M. Alamgir, Abdullah Brooks, Mohamed Jahangir Hossain, Alexander Klimov, Mahmudur Rahman, Stephen P. Luby

**Affiliations:** International Centre for Diarrhoeal Diseases Research, Bangladesh, Dhaka, Bangladesh (E. Azziz-Baumgartner, M. Rahman, A.A. Mamun, R.U. Zaman, P.C. Karmakar, S. Nasreen, S. Mah-E-Muneer, N. Homaira, D.R. Goswami, A. Chakraborty, T. Azim, A. Brooks, M.J. Hossain, S.P. Luby);; Centers for Disease Control and Prevention, Atlanta, Georgia, USA (E. Azziz-Baumgartner, A. Fry, M.-A. Widdowson, J. Bresee, A. Klimov, S.P. Luby);; Institute of Epidemiology, Disease Control and Research, Dhaka (M.S. Haider, B.-N. Ahmed, M.M. Husain, K.M. Jamil, A. Chakraborty, A.S.M. Alamgir, M. Rahman);; World Health Organization, Dhaka (S. Khatun, M. Ahmed)

**Keywords:** Bangladesh, H1N1, influenza, viruses, management, outcome, pandemic (H1N1) 2009, pandemic, respiratory infections

## Abstract

To explore Bangladesh’s ability to detect novel influenza, we examined a series of laboratory-confirmed pandemic (H1N1) 2009 cases. During June–July 2009, event-based surveillance identified 30 case-patients (57% travelers); starting July 29, sentinel sites identified 252 case-patients (1% travelers). Surveillance facilitated response weeks before the spread of pandemic (H1N1) 2009 infection to the general population.

After 2 children in North America were confirmed to have pandemic (H1N1) 2009 infections on April 17, 2009 (*1*), the virus rapidly spread throughout the world. By July 2, 2009, Southeast Asia had reported 1,866 cases (*2*). Officials worried about the effects of pandemic (H1N1) 2009 on the 147,030,000 million population (1,021 persons/km^2^) of Bangladesh (*3*), where 41% of children <5 years of age are underweight (*4*). These concerns prompted Bangladesh to leverage 3 existing surveillance systems (*5*), preparedness plans, and personal protective equipment and oseltamivir stockpiles to guide the response to the pandemic.

During April 2009, Bangladesh enhanced surveillance by implementing border screenings. Upon identification of pandemic (H1N1) 2009 in the general population, Bangladesh encouraged physicians to empirically treat patients who had acute respiratory infection with free oseltamivir if they had risk factors for complications from influenza (i.e., age <5 years or >65 years; diabetes; chronic heart, lung, or liver disease; asthma; neurologic, neuromuscular, hematologic, or metabolic disorders; immune suppression; cancer; obesity; pregnancy; danger signs [rapid, labored or noisy breathing, lethargy, cyanosis, inability to drink, or convulsion], or hospitalization) (*6*). We report the effects of this strategy on a case-series of laboratory-confirmed pandemic (H1N1) 2009 infection identified through enhanced surveillance.

## The Study

**Figure 1:**
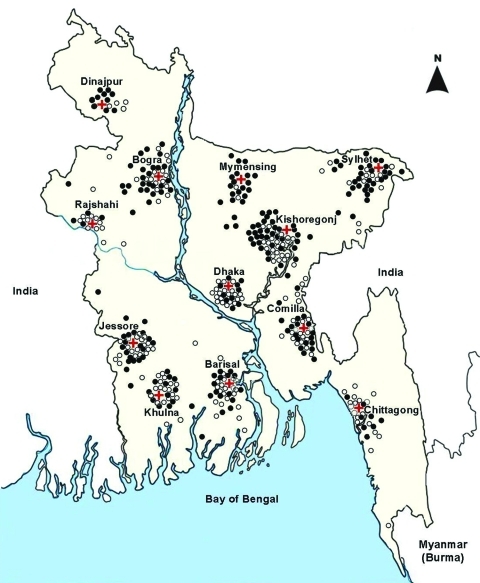
Location of 12 sentinel-site surveillance hospitals (red crosses) and of persons confirmed as infected with pandemic (H1N1) 2009 virus during June 2009–October 2010, Bangladesh. Open circles indicate case-patients identified during 2009; solid circles indicate case-patients identified during 2010. Testing was conducted on the basis of cases of severe pneumonia in hospitalized case-patients (<5 years of age), severe acute respiratory infection in hospitalized case-patients (>5 years of age), influenza-like illness, and acute respiratory infection in ambulatory case-patients (all age groups) identified as part of event-based, sentinel-site, and community-based surveillance systems.

During 2007, Bangladesh started event-based surveillance for the early detection of public health events of international concern. At 6 government and 6 private hospitals (Figure 1), physicians identified >2 epidemiologically linked severe acute respiratory infections, defined as subjective fever within the past 21 days and cough or sore throat (*5*), or severe pneumonia, defined as cough or difficulty breathing, chest in-drawing, stridor while calm, convulsions, inability to drink, lethargy, unconsciousness, or intractable vomiting. During April through November 2009, staff also administered ≈455,000 questionnaires to incoming land and air passengers, contacts, and referrals and collected throat and nasal swab specimens from those who reported cough, sore throat, or shortness of breath and had fever >38°C when assessed with thermal scanners.

During 2007, Bangladesh started sentinel-site surveillance for the early detection of novel influenza. During 2 days per month, physicians collected swab specimens from ambulatory case-patients at hospital clinics with influenza-like illnesses defined as sudden onset fever and cough or sore throat. Physicians also collected swab specimens from children <5 years of age hospitalized with severe pneumonia and person >5 years of age with severe acute respiratory infections (Figure 1).

To explore the epidemiology of seasonal influenza, community-based surveillance began in Bangladesh during 2004. Teams visited an estimated 6,600 preselected households 2×/week to identify acute respiratory infections, defined as the manifestation of 1 major sign (i.e., reported fever; rapid, labored or noisy breathing; lethargy; cyanosis; inability to drink; or convulsion) or 2 minor signs (i.e., cough, rhinorrhea, sore throat, muscle/joint pain, chills, headache, irritability, decreased activity, or vomiting). During 2008, Bangladesh also established a birth cohort of 334 children to explore the potential effects of influenza on their development. Teams visited preselected households 2×/week to identify acute respiratory infections among children <2 years of age. At both sites, teams referred case-patients to physicians who collected nasal wash specimens and provided free care.

Laboratorians tested samples from the 3 surveillance systems for pandemic (H1N1) 2009 virus by using real-time reverse transcription PCR (*7*). Investigators shipped a convenience subset of 28 virus samples to the Centers for Disease Control and Prevention, Atlanta, for antiviral testing and strain characterization.

Investigators described the epidemiology, health-seeking, treatment, and outcome of case-patients who had laboratory-confirmed subtype H1N1 infection by using Pearson χ^2^, Fisher exact, and Wilcoxon rank-sum tests when appropriate. To estimate case-fatality proportion, teams telephoned case-patients or their families >1 month after illness onset.

The Government of Bangladesh conducted enhanced event-based surveillance in the context of emergency response. Ethics committees approved sentinel and community-based surveillance protocols.

**Figure 2:**
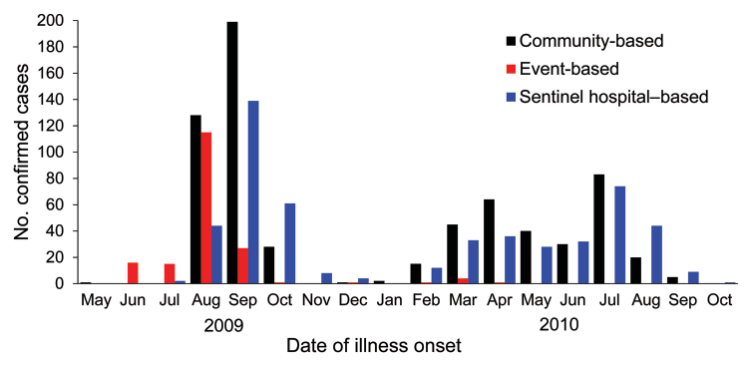
Date of onset of confirmed illness in case-patients with pandemic (H1N1) 2009 by surveillance platform, May 2009–October 2010, Bangladesh.

During June 2009–October, 2010, Bangladesh tested ≈500 passengers, 6 severe acute respiratory infection/severe pneumonia clusters, 5,000 persons identified by sentinel survelliance, and 6,000 persons identified by community-based survelliance and identified 1,371 laboratory-confirmed cases of pandemic (H1N1) 2009 infection (Table 1). During June–July 2009, most (29/30 [97%]) case-patients were identified through event-based surveillance; 17 (63%) were travelers. A rapid increase in the number of sentinel-site case-patients during July 2009 signaled the spread of pandemic (H1N1) 2009 to the general population (Figure 2).

**Table 1:** Demographics of confirmed case-patients with pandemic (H1N1) 2009 virus by surveillance platform, Bangladesh, June 2009–October 2010

Surveillance type	Age, median (range)	No. (%) female patients	No. (%) travelers
Event-based, n = 182	24 y (4 mo–72 y)	71 (39)	51 (28)*
Sentinel-site, n = 527	20 y (1 mo–70 y)	182 (35)†	21 (4)
Kamalapur community-based, n = 621	6 y (3 mo–76 y)‡	303 (49)	0
Mirpur community-based, n = 41	16 mo (3–26 mo)	17 (41)	2 (5)

*Comparison between event-based vs. other surveillance sites, Pearson χ^2^ p<0.0001. †Comparison between sentinel-based vs. other surveillance sites, Pearson χ^2^ p<0.0001. ‡Comparison between Kamalapur-based vs. other surveillance sites, rank-sum p<0.0001.

Isolates from case-patients were antigenically related to A/California/07/2009 (H1N1) and sensitive to oseltamivir. Of the 1,271 case-patients at high risk for complications, 535 (42%) sought treatment within 72 hours of symptom onset, and 7 (3%) of 207 case-patients at sentinel hospitals received oseltamivir (Table 2). The proportion of treatment-eligible case-patients who received oseltamivir decreased from 100% to 0% from June 2009 to October 2010.

**Table 2:** Clinical description of case-patients infected with pandemic (H1N1) 2009 virus seeking treatment, by surveillance platform, Bangladesh, June 2009–October 2010

Description of case-patient	Event-based surveillance, n = 182 (%)	Sentinel-site surveillance, n = 527 (%)	Kamalapur community-based surveillance, n = 621 (%)	Mirpur community-based surveillance, n = 41 (%)
At high risk for complications from influenza illness	79 (43)	331 (63)	285 (4)	41 (100)
Preexisting conditions*	54 (30)†	182 (35)	22 (3)	0
Danger signs (i.e., difficulty breathing or shortness of breath)	50 (27 )†	230 (44)	47 (8)	5( 12)
Treated with oseltamivir‡ when treatment indicated†	4/56 (84)	7/207 (3)	81/272 (30)	1/41 (2)
Median days from symptom onset to treatment with oseltamivir	2 (2–4)	5 (2–8)	4 (1–5)	5
Hospitalization	29 (16)†	259 (49)	0	0
Death	3 (2)†	25 (5)	0	0

*Preexisting conditions among event and sentinel site surveillance case-patients included asthma (71[10%]), chronic obstructive lung disease (31 [4%]), obesity (47 [7%]), immune suppression (20 [3%]), diabetes (12 [2%]), chronic heart disease (10 [1%]), neuromuscular disorders (10 [1%]), liver disease (10 [1%]), and hematologic disorders (8 [1%]), cancer (3 [0.4%]), and pregnancy (3 [0.4%]), while 2 community-based surveillance case-patients had immunosuppression (0.3%), 2 (0.3%) had asthma, and 1 (0.1%) had diabetes. †Comparison between surveillance sites, Pearson χ^2^ p<0.0001. ‡Oseltamivir 5 mg 2×/d for 5 days.

We identified 3 (2%) of 182 event-based and 25 (5%) of 527 sentinel-site decedents (p<0.001) (Table 2). Case-patients who subsequently died, sought treatment a median of 4 days (interquartile range 3–6 days) and received oseltamivir 12 days (interquartile range 5–14 days) after symptom onset compared with 3 days among survivors (p<0.001 and p = 0.01, respectively).

Integration of 3 influenza surveillance systems facilitated response. To delay the spread of the pandemic virus in the general population, Bangladesh used event-based surveillance to identify and treat infected travelers. When sentinel-sites signaled pandemic (H1N1) 2009 among the general population, the government distributed 3.4 million capsules of oseltamivir to hospitals, trained hospital leadership to presumptively treat case-patients with free oseltamivir, and mounted a risk-communication campaign to urge persons at risk for complications to seek care within 3 days of illness development. Officials targeted messages to avoid overwhelming Bangladesh’s hospitals, where there are typically 11 hospitalized patients for every 10 beds (*8*). Meanwhile, officials continued to learn about the epidemiology of pandemic (H1N1) 2009 through population-based surveillance.

Despite government efforts, case-patients sought treatment late, and oseltamivir was underutilized. Less than half (42%) of high-risk patients sought care within 48 hours of disease onset, when oseltamivir is most efficacious. As in other studies (*9*), even severely ill persons who subsequently died were late in seeking treatment. Ill persons frequently were unfamiliar with risk communication messages and may have avoided the expense of seeking treatment. During 2009, only 34% of surveyed households recalled risk communication messages, none could identify oseltamivir (*10*), and those with a history of influenza-like illness paid an average of US $3 when seeking care (i.e., 9% of monthly household expenditure) (*11*).

While the government of Bangladesh provided initial case-patients with oseltamivir, community clinicians provided oseltamivir once pandemic (H1N1) 2009 had spread to the general population. Only a fraction of eligible case-patients then received oseltamivir. Possible explanations for the underutilization of oseltamivir include clinicians’ lack of suspicion of influenza, awareness of treatment guidelines, familiarity with antiviral agents, access to oseltamivir stockpiles, or knowledge of the potential severity of pandemic virus.

Our findings are based on a small case series. Although we identified only 28 decedents, an ongoing study suggests that ≈6,000 persons died as a result of the pandemic (*12*). Nevertheless, we believe that our findings are generalizable to Bangladesh because hospitals were selected as sentinel sites to provide geographically representative data.

## Conclusions

Bangladesh has an effective surveillance system in place for detection of emerging infectious diseases. In spite of timely surveillance, prompt risk communications and free oseltamivir, response may have been hampered by persons’ delays in seeking treatment and by the underutilization of oseltamivir. Our investigation suggests the utility of diverse surveillance systems, the limitations of antiviral drug campaigns, and the importance of influenza prevention through vaccines (e.g., 15 million pandemic [H1N1] 2009 vaccine doses donated to Bangladesh during 2010) and nonpharmaceutical interventions. Such campaigns remain insufficiently used in low-income countries where vaccines are expensive, access to clean water is inadequate, and covering a cough is not customary (*13**,**14*).
